# Gut microbiota, pathogenic proteins and neurodegenerative diseases

**DOI:** 10.3389/fmicb.2022.959856

**Published:** 2022-11-18

**Authors:** Wei Wei, Shixu Wang, Chongchong Xu, Xuemei Zhou, Xinqing Lian, Lin He, Kuan Li

**Affiliations:** ^1^The Mental Hospital of Yunnan Province, Mental Health Center Affiliated to Kunming Medical University, Kunming, China; ^2^School of Forensic Medicine, Kunming Medical University, Kunming, China

**Keywords:** gut microbiota, pathogenic proteins, neurodegenerative diseases, inflammatory factors, dietary therapy, fecal microbiota transplantation

## Abstract

As the world’s population ages, neurodegenerative diseases (NDs) have brought a great burden to the world. However, effective treatment measures have not been found to alleviate the occurrence and development of NDs. Abnormal accumulation of pathogenic proteins is an important cause of NDs. Therefore, effective inhibition of the accumulation of pathogenic proteins has become a priority. As the second brain of human, the gut plays an important role in regulate emotion and cognition functions. Recent studies have reported that the disturbance of gut microbiota (GM) is closely related to accumulation of pathogenic proteins in NDs. On the one hand, pathogenic proteins directly produced by GM are transmitted from the gut to the central center *via* vagus nerve. On the other hand, The harmful substances produced by GM enter the peripheral circulation through intestinal barrier and cause inflammation, or cross the blood–brain barrier into the central center to cause inflammation, and cytokines produced by the central center cause the production of pathogenic proteins. These pathogenic proteins can produced by the above two aspects can cause the activation of central microglia and further lead to NDs development. In addition, certain GM and metabolites have been shown to have neuroprotective effects. Therefore, modulating GM may be a potential clinical therapeutic approach for NDs. In this review, we summarized the possible mechanism of NDs caused by abnormal accumulation of pathogenic proteins mediated by GM to induce the activation of central microglia, cause central inflammation and explore the therapeutic potential of dietary therapy and fecal microbiota transplantation (FMT) in NDs.

## Introduction

Neurodegenerative diseases (NDs) include Alzheimer’s disease (AD), Parkinson’s disease (PD), Huntington’s disease (HD), and multiple sclerosis (MS) are characterized by associated neuron damage resulting from a buildup of neurotoxic substances in the brain (Zhang et al., 2022). And as the world’s population ages, so will the burden of NDs (Cottler et al., 2015; Ravindranath et al., 2015). The abnormal accumulation of pathogenic proteins is considered to be common feature of NDs, these abnormal accumulations can occur in a variety of aggregates, including naturally unfolded monomers, β-rich oligomers or fibrils, and stable pathogenic proteins fibrils (Lashuel et al., 2002; Volles and Lansbury, 2003). Tau protein, amyloid β-protein (Aβ) and α-synuclein (α-syn) abnormal accumulations have been widely reported to be associated with the occurrence of NDs (Giacomelli et al., 2017; Ryder et al., 2022). The abnormal accumulation of these pathogenic proteins can over-activate astrocytes and microglia leading to a range of neuron and synaptic plasticity damage, etc., and that eventually led to the development of NDs (Parhizkar and Holtzman, 2022; Zhu et al., 2018).

In recent years, microbial infections was regard as a risk factor for NDs (Lotz et al., 2021). Gut microbiota (GM) have been reported to be related to the occurrence and development of NDs (Fang et al., 2020; Sun et al., 2020; Singh et al., 2021). The GM is thought to be critical for brain physiological processes such as myelination, synaptic plasticity, neurogenesis and glial cell activation and regulate mental processes such as emotion and cognition (Diaz Heijtz et al., 2011; Pellegrini et al., 2018). GM can directly or indirectly affect the abnormal accumulation of pathogenic proteins in the brain. On the one hand, GM can directly cause the generation of pathogenic proteins by acting on the gut and enter the brain through the vagus nerve (Kim et al., 2019). On the other hand, GM can trigger a cytokine storm that causes abnormal accumulation of pathogenic proteins in the brain by causing an excessive inflammatory response in the body (Megur et al., 2020). And, these abnormal accumulation of pathogenic proteins of different origins trigger a more severe inflammatory and disease response. Therefore, the mechanism of abnormal aggregation of pathogenic proteins in NDs directly or indirectly mediated by GM in the brain is expected to provide a theoretical basis for suppressing the accumulation of pathogenic proteins in NDs by intestinal microorganisms in the future.

GM dysregulation is an important factor in the occurrence and development of nervous system diseases (Ghezzi et al., 2022). Therefore, GM targeting is expected to become a new treatment for NDs, with great clinical research prospects. At present, studies have proved that diet, antibiotics, stress, lifestyle and surrounding environment are all factors affecting the composition of GM (Sorboni et al., 2022). In this review, in addition to reviewing the relationship between GM and pathogenic proteins in NDs, two current approaches based on modulation of host GM to ameliorate the pathological changes in NDs, namely dietary therapy and FMT, will be described. It provides new ideas for future research on clinical treatment strategies of NDs.

## Is there a relationship between gut microbiota and pathogenic proteins of neurodegenerative diseases?

In the past decade, it has been widely reported that GM not only directly affect the host gut environment, but also indirectly affect the health of the host. GM is a complex community of microorganisms colonizing the digestive tracts of humans. It consists of more than 1,500 species, including more than 50 different phyla (Yang et al., 2020). Among them, *Bacteroidetes* and *Firmicutes* are the most important, followed by *Proteobacteria*, *Fusobacteria*, *Tenericutes*, *Actinobacteria* and *Verrucomicrobia*, accounting for 90% of the total microbial population in humans (Gomaa, 2020). In a healthy state, the GM is in ecological balance, showing a high diversity and rich microbial population, which plays a variety of important roles in host, such as digestion, metabolism, immune regulation, anti-aging and emotional cognition (Vera-Urbina et al., 2022).

Many studies have shown that there is a close correlation between the GM and the brain, which can communicate with each other directly and indirectly, including neural, immune and endocrine pathways (Cryan et al., 2019). This links the gut to a central brain region that controls emotion and cognition (Kesika et al., 2021). Therefore, the important concept of microbial-gut-brain (MGB) axis was proposed, and it has become a research hot spot in the field of neuroscience, even though the mechanism involved is not completely clear (Maiuolo et al., 2021). The GM plays a key role in early brain development and adult neurogenesis. It is well known to have a significant impact on NDs (Kowalski and Mulak, 2019; Nagu et al., 2021). GM dysbiosis and changes in the function and structure contribute to the development of NDs, such as PD and AD (Pistollato et al., 2016). Several studies now suggest a potential link between GM, neuroinflammation, and cognitive impairment. GM changes precede amyloidosis and neuroinflammation (Chen et al., 2020). Cognitive impairment and amyloid deposition are associated with increased abundance of pro-inflammatory bacteria (e.g., *Escherichia*, *Shigella*) and decreased abundance of anti-inflammatory bacteria (e.g., *Bacteroides fragilis*) (O'Toole and Jeffery, 2015; Rogers et al., 2016; Sharon et al., 2016). Furthermore, certain gut microbial components may be actively involved in regulating neuroinflammation and protein misfolding (González-Sanmiguel et al., 2020; Parker et al., 2020). GM have been shown to produce amyloid, lipopolysaccharide (LPS), or other biological substances that disrupt intestinal homeostasis, affect the central nervous system through the MGB axis, and mediate the pathological process of NDs, such as inflammation, amyloid, and Tau protein accumulation in the brain (Friedland, 2015; Xu and Wang, 2016). Among them, extracellular bacterial amyloid produced by *Pseudomonas*, *Salmonella*, and *EScherichia coli* (*E*. *coli*) may contribute to the pathological process of α-syn and inflammatory responses in the gut and brain, or may mediate the formation of Aβ amyloid through the blood–brain barrier (BBB), leading to NDs (Needham et al., 2020; Sampson et al., 2020; Zhang et al., 2022).

For AD, A study of 40 AD patients also showed increased abundance of the proinflammatory bacteria *Escherichia* and *Shigella*, and decreased abundance of the anti-inflammatory bacteria *E*. *rectale*, compared with healthy people and cognitive patients without amyloid, and has been suggested to be associated with peripheral inflammatory states. The relationship between them and amyloidosis is worth further investigation (Cattaneo et al., 2017). Endotoxins produced by GM may be involved in the inflammatory and pathological processes associated with amyloidosis and AD. After long-term injection of LPS into the fourth ventricle of rats, inflammatory responses and AD pathological features can be seen in the brain. In addition, *in vitro* studies have found that *E*. *coli* endotoxin can enhance the formation of Aβ fibrin, which may be involved in the formation of pathogenic amyloid protein and inflammatory response in the brain of AD patients (Blanco et al., 2012). Interestingly, antimicrobial-induced intestinal dysbiosis can exacerbate neuroinflammation and amyloid deposition in AD models, while symptoms and pathological processes of amyloid deposition in brain were alleviated in AD mice transplanted with healthy mice or healthy human intestinal bacteria (Asti and Gioglio, 2014). In another mouse study, GM dysregulated pregnancy was vertically transferred to offspring after antibiotic treatment, similarly demonstrating that specific GM ameliorated memory impairment and reduced Aβ aggregation in preclinical AD models (Bello-Medina et al., 2022). In other studies, chronic *H*. *pylori* infection has been shown to affect AD by releasing a large number of inflammatory mediators. Plasma levels of β-amyloid peptide 1–40 and 1–42 were increased in AD patients infected with *H*. *pylori*. Furthermore, *H*. *pylori* filtrate can induce AD Tau hyperphosphorylation. Treatment with probiotics, such as *Lactobacillus* and *Bifidobacterium*, reduced amyloid beta formation and improved cognitive function (Nimgampalle and Kuna, 2017; Sochocka et al., 2019).

In PD, One clinical study showed that the fecal abundance of *Prevotellaceae* decreased by 77.6% in PD patients compared with healthy volunteers, while the abundance of *Enterobacteriaceae* increased, and was positively correlated with the severity of the patients’ dyskinesia (Scheperjans et al., 2015). Forsyth et al. (2011) also found that PD patients had an increase in intestinal permeability accompanied by an increase in *Enterobacteriaceae* abundance in the gut and an increase in pathological α-syn in the gut and brain. Furthermore, GM has been found to promote pathogenic α-syn accumulation, neuroinflammation, dopamine neuronal degeneration, and motor dysfunction in PD mouse models (Sampson et al., 2016). It may be mediated by substances produced by GM. Recent studies have found that some GM-derived amyloid proteins can cause abnormal changes of α-syn in gut and brain, leading to motor dysfunction (Sampson et al., 2020). Therefore, GM is closely related to NDs related pathogenic proteins. However, questions remain about how GM-derived amyloid affects NDs, In the future, the relationship between intestinal and brain pathological proteins and the specific mechanism of action need to be further studied.

## Gut microbiota affects the accumulation of pathogenic protein in neurodegenerative diseases by direct and indirect pathways

**Figure 1:**
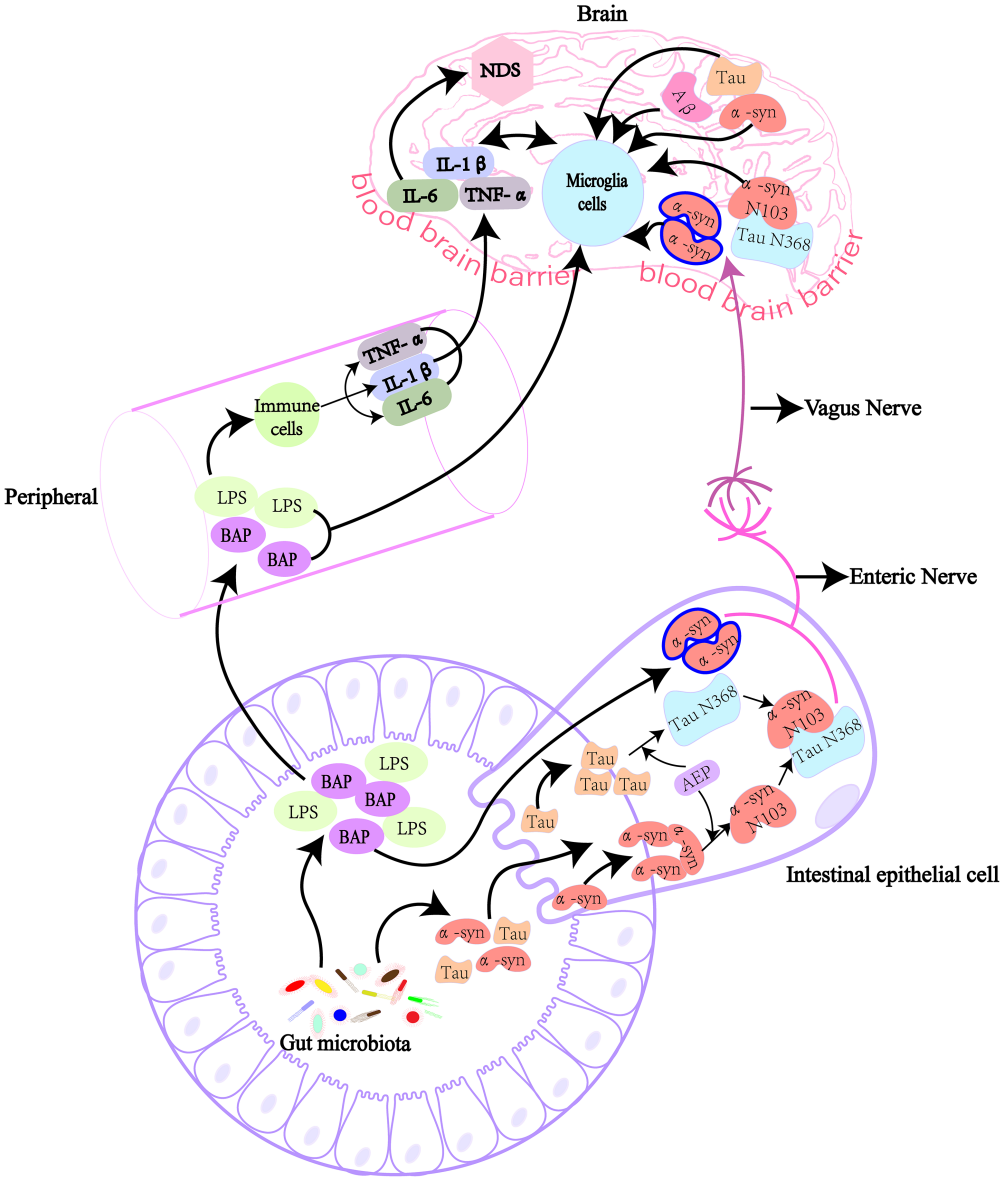
GM affect the production of pathogenic proteins in the brain directly and indirectly. GM can produce lipopolysaccharide and amyloid protein, and these harmful substances enter the body and cause NDs when intestinal and blood-brain barriers are damaged. Bacterial amyloid directly cause the accumulation of pathogenic α-syn in the gut in a “cross-seeding” manner, or the intestinal AEP cleaved α-syn and Tau proteins at N103 and N368 sites, respectively, to form α-syn N103/Tau N368 complex. These pathogenic proteins then enter the brain through the vagus nerve, causing cognitive dysfunction. Bacterial amyloid and lipolysaccharide pass through the damaged intestinal barrier and blood-brain barrier to cause inflammation in the peripheral circulatory system, or cause inflammation in the brain to produce pro-inflammatory cytokines, indirectly leading to the synthesis and deposition of pathogenic amyloid proteins. LPS, lipopolysaccharide; α-syn, α-synuclein; Aβ, β-amyloid; AEP, asparagine endopeptidase; IL, interleukin; TNF-α, tumor necrosis factor-α; BAP, bacterial amyloid protein.

The effects of GM on pathogenic proteins are complex. In general, GM is closely related to NDs. Bacterial amyloid proteins, LPS and other components of GM can trigger NDs through a variety of ways. We reviewed previous studies and summarized them into two possibilities. One possibility is that it is taken up by the intestinal epithelium and then retrograde transported to the central nervous system through the vagus nerve. The Braak hypothesis (Braak et al., 2003). Another possibility is to enter the brain through damaged intestinal and BBB, causing corresponding pathophysiological processes (Desplats et al., 2009).Therefore, we explored the immune and vagal pathways will be used to elucidate the relationship between GM, pathogenic Protein and NDs (Figure 1).

### Gut microbiota directly stimulates production of pathogenic proteins

NDs are associated with abnormal aggregates of pathogenic proteins, of which humans encode about 30, while GM also produce functional pathogenic proteins, such as curli, Tau, Aβ, α-syn and FapC (Kesika et al., 2021; Wang et al., 2021). Related studies have shown that amyloid produced by GM may lead to the accumulation of other pathogenic proteins with different structures through “cross-seeding,” which leads to the misfolding of neuronal proteins (Blanco et al., 2012; Friedland and Chapman, 2017). For example, a variety of intestinal strains can express Curil protein, such as *E*. *coli*, *S*. *Typhimurium*, *Citrobacter SPP* and *Enterobacter SPP* (Zogaj et al., 2003; Dueholm et al., 2012). Sampson et al. (2020) found that curli protein-producing *E*. *coli* can promote α-syn pathology and inflammation in the gut and brain of mice, and purified CsgA of curli protein subunit can accelerate α-syn aggregation *in vitro*. When CsgA of curli subunit of *E*. *coli* was knocked out or inhibited, α-syn-induced cell death was significantly reduced, and mitochondrial and neuron functions were restored (Wang et al., 2021).

What’s more, In PD patients, accumulation of pathogenic α-syn is observed in the intestinal tissue, which is thought to occur before the onset of gastrointestinal motor symptoms (Hilton et al., 2014). Pathogenic α-syn was found in both enteroendocrine cells and intestinal neurons. It could reach the brain from the intestine through the vagus nerve and cause pathological changes in the central nervous system (Holmqvist et al., 2014; Chen and Lin, 2022). The study found that pathogenic α-syn was injected into the duodenum and pylorus muscle layer in mice produced symptoms similar to those of PD and successively in the vagus nerve dorsal motor nucleus, after brain tail, the basolateral amygdala, dorsal raphe nucleus and substantia nigra compacta found pathogenic α-syn, and cut off the vagus nerve can inhibit the occurrence of this phenomenon (Kim et al., 2019). A cohort study of vagotomy in PD patients also found a reduction in PD risk after total trunk vagotomy (Svensson et al., 2015). In addition, the pathological development of α-syn from the gut to the brain is age-related, and older mice are more susceptible than younger mice (Challis et al., 2020). More severe forms of disease can occur in the gut, asparaginyl endopeptidase (AEP) cleaved α-syn and tau protein at N103 and N368 sites, respectively, and produced α-syn N103 and Tau N368 in intestinal tract. α-syn N103 interacts with tau N368 to promote each other’s fibrosis and transmission from the gut to the brain, triggering the loss of dopaminergic neurons in the substantia nigra. And the complex spreads faster and causes more severe disease than the normal form of α-syn (Ahn et al., 2020). Abnormal accumulation of α-syn was found in the appendix of patients with PD and appendectomy can affect the incidence of PD (Chen et al., 2021). Therefore, the gut-derived α-syn play an important role in the development of NDs. the vagus nerve is involved in the transmission of intestinal pathogenic α-syn to the brain. However, the underlying mechanism of how the pathogenic α-syn is transmitted to the brain *via* the vagus nerve has not been clarified.

Simultaneously, activation of the CCAAT/ EBPβ/AEP pathway in the gut and brain was also found in AD patients, inducing Aβ and Tau fiber formation and propagation to the brain *via* the vagus nerve (Chen et al., 2021). The GM of the aged AD mice transfer to the young mice, which can activate the CCAAT/ EBPβ/ AEP pathway in the brain of the young mice and accelerate the progression of AD. Moreover, prebiotic-enriched *Lactobacillus salivarius* can significantly inhibit the CCAAT/ EBPβ/ AEP pathway and reduce generation of Tau protein and oxidative stress (Chen et al., 2020). And, microbial prions in the human microbiome may also be involved in protein misfolding that initiates NDs (Flach et al., 2022). The Aβ from gut may also be involved in abnormal accumulation in the brain. After Aβ 1–42 oligomer was injected into the gastrointestinal tract of mice, Aβ migrated from the gastrointestinal tract to the vagus nerve and brain after 1 year, and induced intestinal dysfunction and cognitive impairment in mice (Sun et al., 2020). GM may through the gut -derived pathogenic proteins take part in the development of NDs.

### Gut microbiota indirectly stimulates production of pathogenic proteins

The gut mucosal lymphatic tissue also has 70 to 80% of the body’s immune system, so it is considered to be the largest and most important immune organ in the body (Hooper et al., 2012). These lymphatic tissues maintain continuous close contact with the human GM. GM are known to secrete a range of compounds, such as LPS and amyloid. It has been suggested that LPS and amyloid can cause NDs by directly passing through the damaged gastrointestinal tract and BBB, or indirectly through these protective physiological barriers by LPS and amyloid triggered cytokines or other pro-inflammatory factors (Kesika et al., 2021). In addition, these compounds may also can increase the permeability of the intestinal barrier, damage the immune system, increase the production of proinflammatory cytokines (e.g., IL-6, IL-1β, and TNF-α), and further enhance the gut and the permeability of BBB, significantly increased inflammatory response. Induced excessive synthesis and accumulation of Aβ and α-syn amyloid proteins, and the Tau protein phosphorylation, eventually leading to neurodegeneration (Quigley, 2017; Sochocka et al., 2019; Zhao et al., 2019; Rosario et al., 2021). Bacterial amyloid can interact with Toll-like receptor 2 (TLR2) to activate NF-κB signal and COX-2, promote the production of proinflammatory cytokines, such as IL-17A and IL-22, and the production of amyloid in brain neurons (Nishimori et al., 2012; Kesika et al., 2021).

Lipopolysaccharide is a major component of the cell wall of Gram-negative bacteria and an endotoxin. When bacteria invade the body, they release LPS. Recent studies have shown that high levels of Aβ are present in brain tissues after LPS intervention. LPS first binds to lipopolysaccharide binding proteins to transport LPS to the membrane surface of immune cells, it binds to a protein on the surface of the membrane, CD14, which then transport LPS to toll-like receptor 4 (TLR4) and myeloid differentiation protein 2 (MD2) protein complex. As a special exocrine protein, MD2 helps TLR4 recognize LPS. When LPS is combined with TLR4 extracellular groups, the conformation of the intracellular groups changes, and the signal transduction into immune cells activates signal molecules such as MyD88, IL-1R-associated protein kinase (IRAK), IRAK2 and tumor necrosis factor receptor-associated factor 6 (TRAF6). Through a series of biochemical reactions, It phosphorylates the inhibitory protein IB kinase (IK) complex, degrades IκB, and eventually activates the transcription factor NF-κB, which crosses the nuclear membrane and binds to specific regions on chromosomes, promotes the expression of multiple cytokines, and causes systemic inflammation, resulting in increased Aβ levels and neuronal death, ultimately leading to cognitive impairmen (Milosevic et al., 2019; Zhao et al., 2019; Kesika et al., 2021). Morever, LPS enters the body through the damaged intestinal barrier, causing an inflammatory response and increasing the expression of amyloid precursor protein (APP). APP is a transmembrane glycoprotein that plays an important role in the pathogenesis of AD. At the same time, promotion of β/γ-secretase will abnormally cleat APP and induce the production of Aβ (González-Sanmiguel et al., 2020). Similarly, in PD, GM ecology is dysregulated, causing elevated levels of LPS, systemic inflammation through the TLR4/NF-κB pathway, disruption of the BBB, and triggering α-syn accumulation (Sorboni et al., 2022). Moreover, LPS has been suggested to be a key mediator of α-syn aggregation in the enteric nervous system (Bhattacharyya and Bhunia, 2021).

In addition, proteins of bacterial origin (e.g., curil, Aβ, and α-syn) produced by intestinal microorganisms were found to initiate the accumulation of Aβ peptide in AD (Friedland and Chapman, 2017. This process may be mediated by the innate immune system or cross-seeding (Kesika et al., 2021; Chidambaram et al., 2022). Bacterial amyloid protein has been identified as pathogen-associated molecular pattern (PAMP), and its communication messengers include TLR1/2, CD14, NF-κB and iNOS (Chen et al., 2016). Therefore, these results suggest that the immune pathway serves as an important bridge between GM and NDs-related pathogenic proteins.

## Pathogenic proteins activates microglia to produce inflammatory factors

**Figure 2:**
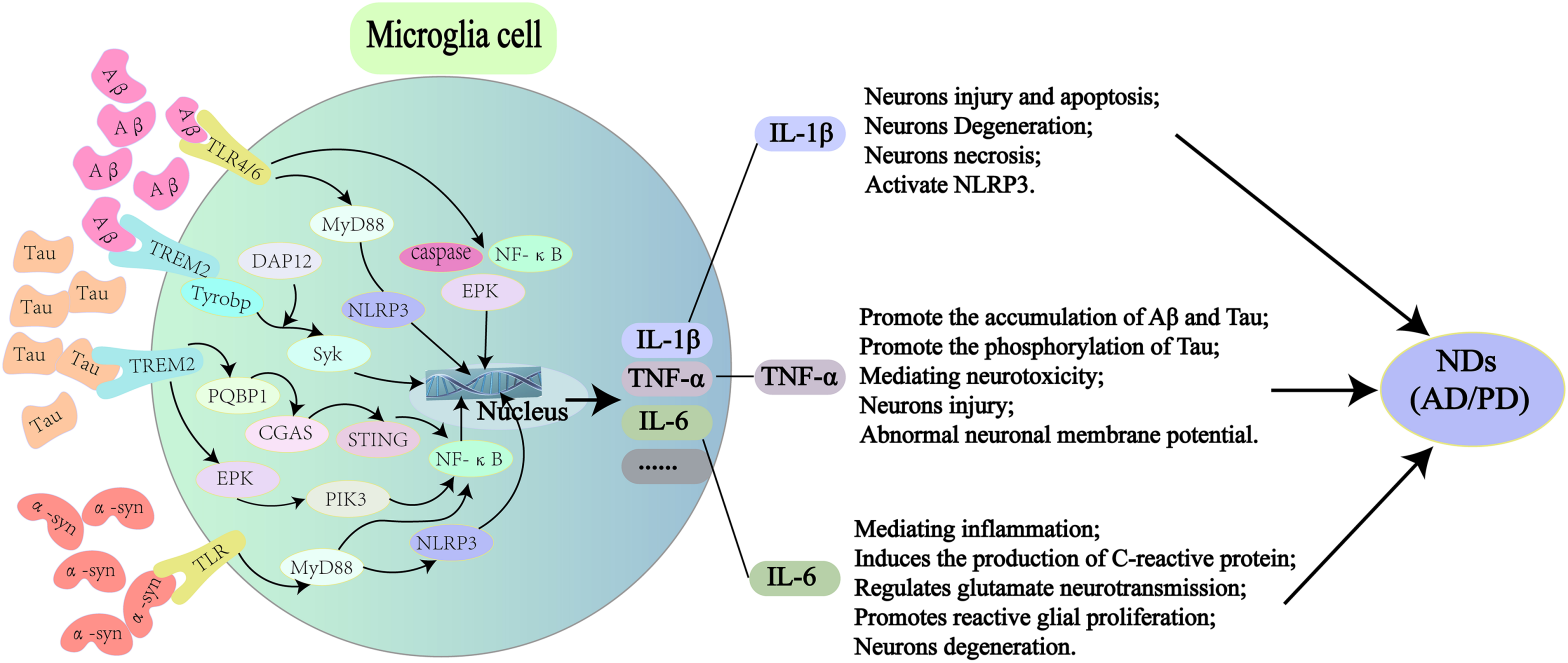
Pathogenic proteins activate signal transduction pathways in microglia leading to NDs. In AD, Aβ can bind to TLR4/6 on microglia and induce the release of inflammatory factors (IL-1β, TNF-α, and IL-6) through the downstream MyD88/NLRP3 and NF-κB signaling pathways. It also activates TREM2 on microglia, causing DAP12 to enhance Syk and induce a cascade of protein tyrosine phosphorylation, which leads to apoptosis and myelin damage through immune pathways. For Tau protein, it can activate microglia through PQBP1/CGAS/STING pathway, and 40% NF-κB can amplify inflammatory response in this process, leading to a series of nerve damage. The rest is supplemented by TREM2/ERK/PIK3. In PD, α-syn can stimulate microglia to secrete proinflammatory cytokines, such as ROS, TNF-α, IL-1β, COX2, and iNOS through TLR/MyD88/NLRP3 pathway. It also binds to TLR2 to induce nuclear translocation of NF-κB, which triggers the production of NLRP3, pro-IL-1β and pro-IL-18, and ultimately IL-1β secretion. TLR, toll-like receptors; MyD88, myeloid differentiation factor 88; NLRP3, NOD-like receptor thermal protein domain associated protein 3; NF-κB, nuclear factor kappa-B; TREM2, triggering receptor expressed on myeloid cells 2; DAP12, TYRO protein tyrosine kinase binding protein; PQBP1, polyglutamine binding protein-1; CGAS, cyclic GMP-AMP synthase; STING, interferon gene stimulation protein; ERK, extracellular regulated protein kinases; PIK3, phosphatidylinositide 3-kinases; ROS, reactive oxygen species; COX2, cyclooxygenase 2; iNOS, inducible nitric oxide synthase.

Glial cells in the brain include astrocytes, oligodendrocytes, and microglias. Microglia account for 5–15% of the total brain cells (Pelvig et al., 2008). As central scavenger cells, microglia play an important role in central immune defense and the regulation of immune microenvironment. Microglia are closely related to central NDs, such as AD, PD and Amyotrophic lateral sclerosis (ALS) (Harms et al., 2021; Leng and Edison, 2021; Vahsen et al., 2021; Figure 2).

### Aβ in Alzheimer’s disease

Studies have shown that a variety of receptors on microglias mediate NDs caused by pathogenic proteins, such as class B scavenger receptor CD36, integrin-associated protein/CD47, and α_6_β_1_-integrin (Bamberger et al., 2003) through pattern recognition receptors (PRR) of the innate immune system (Stewart et al., 2010). Peptide interactions of Aβ fibrils receptors with CD36, scavenger receptors, CD47, and α_6_β_1_ integrin inhibit Aβ-stimulated Tyr kinase-based signaling cascades in THP-1 monocytes and mouse microglias, as well as IL-1β production (Bamberger et al., 2003). Aβ fibrils can trigger microglial inflammation *via* Toll-like receptors4/6 (TLR4/TLR6) (Stewart et al., 2010). Mice with TLR4 mutation were hybridized with AD transgenic mice and showed more Aβ plaque deposition in the center (Tahara et al., 2006). Activation of Aβ by caspase and signal-dependent transcription factors (such as NF-κB and AP-1) leads to the production of a large number of inflammatory cytokines, such as IL-1β, TNF-α, and IL-6, which may act on neurons to induce apoptosis (McCoy and Tansey, 2008). In addition, cytokines (such as TNF-α and IL-1β) released by microglias can activate astrocytes, and cytokines released by astrocytes can lead to further activation of microglias (Saijo et al., 2009). Aβ also promotes Nod-like receptor (NLR) family pyrin domain containing 3 (NLRP3) inflammasome activation and IL-1β secretion by acting on TLR/MyD88 (Terrill-Usery et al., 2014; Friker et al., 2020). A recent study showed that Aβ acts on the trigger receptor 2 (TREM2) expressed on bone marrow cells on microglia, which transmits intracellular signals through an associated adapter, DAP12, which enforces A protein tyrosine kinase, Syk, leading to a cascade of protein tyrosine phosphorylation. This cascade reaction can lead to the differentiation of microglia into disease-associated microglia, which ultimately causes cell apoptosis and myelin sheath injury through immune pathways (Ellwanger et al., 2021).

### Tau in Alzheimer’s disease

In addition to Aβ, Tau is also an important pathogenic protein in AD. A recent study showed that Tau can activate microglia through the PQBP1-CGAS-STING pathway (Jin et al., 2021), and the activated microglia can produce IL-1β, TNF-α, IL-6 and other inflammatory factors through NF-κB transcription factor, ultimately leading to cognitive impairment in mice (Wang et al., 2022). TREM2 has been shown to be a risk factor for AD (Guerreiro et al., 2013), and activation of microglia in the brain of mouse AD models and human AD patients can occur through TREM2-dependent and TREM2-independent mechanisms (Keren-Shaul et al., 2017), and NF-κB activation can also be induced by TREM2 signaling (Zhong et al., 2017). Jin et al. (2021) showed that Tau activated 40% of NF-κB *via* the PQBP1-CGAS-STING pathway and TREM2-ERK/PIK3 pathway complemented NF-κB activation in the PQBP1-CGAS-STING pathway. A recent interesting study found that Tau can produce phosphorylated modification in microglia after being phagocytic by microglia, and phosphorylated Tau can enhance Tau diffusion by secreting vesicles and aggravating central inflammatory response (Clayton et al., 2021). Another study also showed that, Transmission of Tau pathology depends on the underlying microglia circuit, supporting the hypothesis that neuronal transmission of Tau is induced by activated microglia (Pascoal et al., 2021). An *in vitro* and *in vivo* study showed that Tau aggregation can activate NLRP3-ASC inflammatory bodies of microglia and aggravate endogenous and non-exogenous Tau pathology *in vivo* (Stancu et al., 2019).

### α-Syn in Parkinson’s disease

Recent studies have shown that the serum and cerebrospinal fluid of PD patients have proinflammatory characteristics, suggesting cytotoxic microglial activity (Brodacki et al., 2008). Microglia and misfolded α-syn are thought to be involved in a vicious cycle: α-syn itself can elicit immune responses, since both secreted and aggregated α-syn are known to activate microglia, and inflammatory mediators can promote α-syn aggregation (Zhang et al., 2005; Blandini, 2013). α-syn promotes NLRP3 inflammasome activation through TLR/MyD88 (Daniele et al., 2015), and promotes the production of ROS, TNF-α, IL-1β, COX2, iNOS and other inflammatory factors in microglia (Su et al., 2008; Watson et al., 2012). Meanwhile, studies have shown that FLZ, a novel phosphamide derivative, can improve the inflammatory state of the brain in PD mouse models by inhibiting TLR4/MyD88/NF-κB signaling pathway (Zhao et al., 2021a). α-syn fibrils, but not monomers, activate the NLR family Pyrin domain containing NLRP3 inflammasome and induce IL-1β and cleaved Caspase-1 production and release. Pretreatment of mouse microglia with the small molecule NLRP3 inhibitor MCC950 improved α-syn mediated inflammatory response (Pike et al., 2021). Studies have shown that α-syn protein can lead to the production of NLRP3, pro-IL-1β and pro-IL-18 through the “α-syn-TLRS-NF-κB /NLRP3 inflammasome axis.” First, α-syn binds to TLR2, and then induces the downstream nuclear translocation of NF-κB, which triggers the production of NLRP3, pro-IL-1β and pro-IL-18, and eventually leads to the production of cytokines such as IL-1β (Li et al., 2021). In a recent study of primary human microglia, α-syn protein was found to activate the secretion of IL-1β by NLRP3 in microglia (Pike et al., 2021).

## Inflammatory factors and neurodegenerative diseases

Elevated IL-1β levels are often observed in NDs (Heneka et al., 2018). IL-1β plays an important role in the central nervous system, and many cells in the central nervous system can express the IL-1β receptor, which can cause an inflammatory signaling cascade, ultimately leading to neuron injury and cell death (Allan et al., 2005). Apoptosis and necrotizing apoptosis are different modes of cell death that have been shown to promote neuroinflammation and neuronal degeneration in a variety of NDs, including multiple sclerosis (Zhang et al., 2017; Yuan et al., 2019). Microglia are activated around amyloid plaques in AD, and the activated microglia produce IL-1β, which can lead to neuronal degeneration (Griffin and Mrak, 2002). The typical mechanism of IL-1β production by microglia involves activation of NLRP3 inflammasome (Mendiola and Cardona, 2018; Place and Kanneganti, 2018), a multi-molecular scaffold whose primary function is to sense the amplification and propagation of pro-inflammatory signals from one cell to another by driving cytokine secretion. The three traditional components of NLRP3 inflammasome are: (1) intracellular pattern recognition receptor NACHT domain, leucine-rich repeat (LRR), and NLRP3 (Heneka et al., 2014); (2) ASC consists of a Pyrin domain and a caspase activation and recruitment domain (CARD) (Latz et al., 2013); (3) Caspase-1 (Cysteine-aspartate protease) (Boucher et al., 2018). In the APP/PS1 mouse model, NLRP3 inflammasome cleaves immature pro-IL-1 to produce mature IL-1β, which mediates neuronal damage and cognitive dysfunction (Heneka et al., 2013).

Tumor necrosis factor -α (TNF-α) is a 25 kDa transmembrane protein that can be produced by a variety of cells, including microglia (Parameswaran and Patial, 2010). TNF-α binds to tumor necrosis factor receptor (TNFR1/2) and plays a variety of downstream roles, including immune stimulation, resistance to infectious agents, malignant cell cytotoxicity, sleep regulation and embryonic development (Idriss and Naismith, 2000). TNF-α-mediated inflammation can also lead to Aβ plaques and Tau protein accumulation in the brain of AD patients (Baj and Seth, 2018), and phosphorylated Tau can further aggravate the central inflammatory response (Clayton et al., 2021). Production of TNF-α in microglia stimulated by amyloid over time induces neuroinflammatory responses associated with muscular atrophy in AD, PD, and MS (Baj andSeth,2018). TNF-α inhibitors reduce central Aβ and Tau levels and improve cognitive dysfunction in a mouse model of dementia (Shi et al., 2011). In a clinical study, curcumin reduced TNF-α and may reduce the accumulation of Aβ plaques and Tau in the hypothalamus, and was also found to improve cognitive dysfunction (Small et al., 2018). TNF-α can enhance n-methyl-D-Aspartate (NMDA) receptor, mediate neurotoxicity, increase glutamate, and cause nerve cell damage (Zou and Crews, 2005). In addition, TNF-α can also affect neuronal membrane potential, resulting in long-term disturbance of intracellular Ca^2+^ balance and abnormal cell function (Choi et al., 2002).

IL-6 is a polypeptide composed of α and β chains (Kaur et al., 2020). The change in central IL-6 concentration is mainly due to the response of astrocytes and microglias to inflammation (Guo et al., 2021). Dysregulation of IL-6 is associated with various cognitive dysfunction, and individuals with high levels of IL-6 in the blood are at a higher risk of cognitive impairment than individuals with low Levels of IL-6 (Bradburn et al., 2017). IL-6 acts as an important proinflammatory cytokine and a key mediator of the acute phase response, and IL-6 induces c-reactive protein (CRP) production. Typical IL-6 signaling involves the binding of IL-6 to its homologous receptor, causing the formation of a dimer of GP130 protein, Intracellular signal transduction and transcriptional regulation are then activated through the Janus kinase/signal transduction and transcriptional activator (Jak/STAT) pathway and the transcription factor cytokine signal transduction inhibitor 3 (SOCS3). Another signaling pathway is IL-6 binding to IL-6R to produce soluble SIL-6R, which then activates GP130 signaling (Dugan et al., 2009). Increased levels of central IL-6 have been shown to mediate disease behaviors, including lethargy, insanity, and cognitive deficits (Cartmell et al., 2000). Newest study suggests that central IL-6 also regulates glutamate neurotransmission (Qiu and Gruol, 2003), and it has been found that IL-6 in transgenic mice promotes reactive glial proliferation and further promote neuronal degeneration in acute response (Chiang et al., 1994). Therefore, this storm of inflammatory actived by glial cells is an important contributor to NDs.

## Neurodegenerative diseases newly treatment

**Figure 3:**
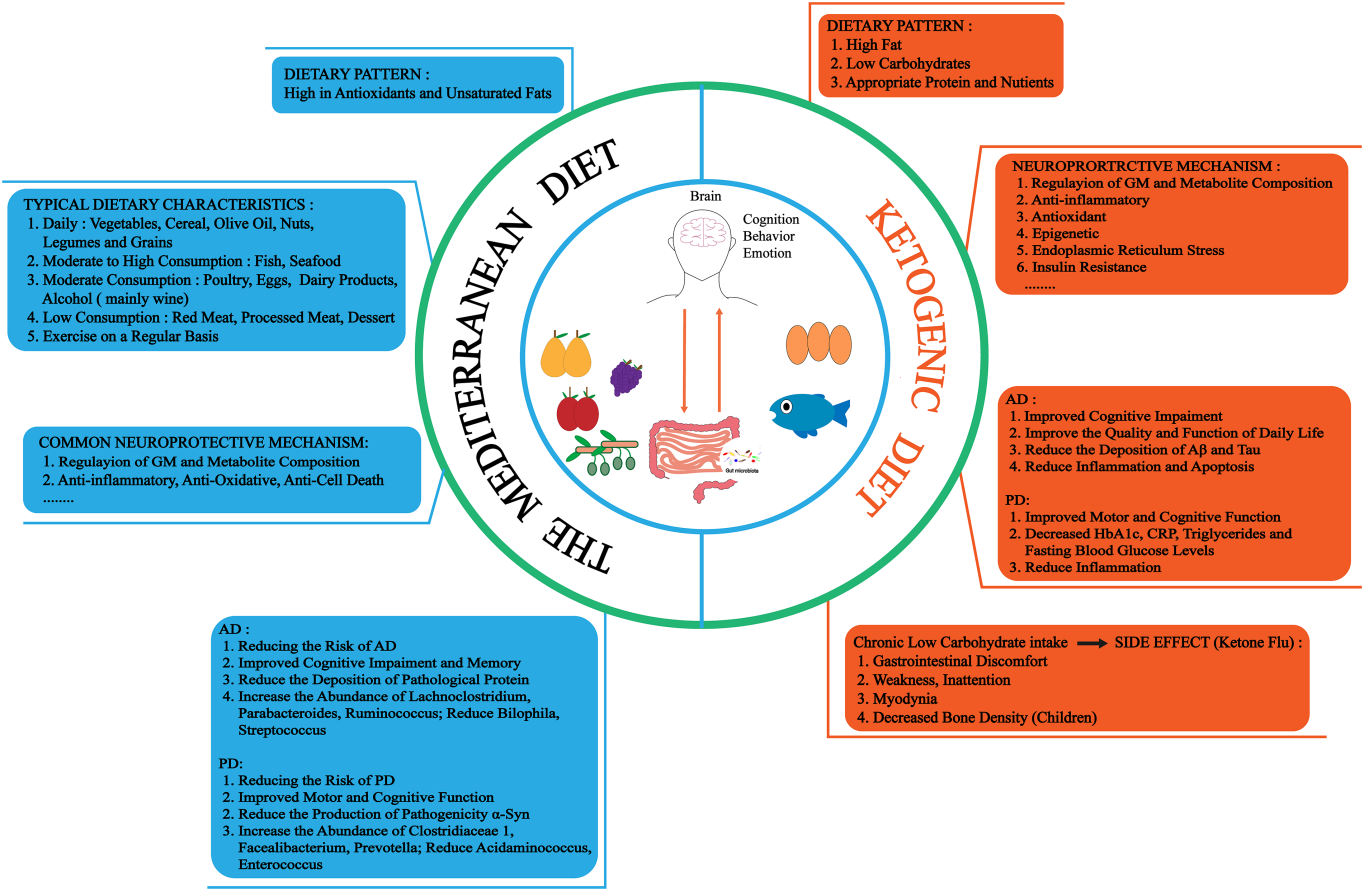
There are two dietary treatments for NDs, the Mediterranean diet (MedD) and the ketogenic diet (KD). MedD is a dietary pattern rich in antioxidants and unsaturated fatty acids, which has neuroprotective effects such as regulating GM and metabolite composition, anti-inflammatory, antioxidant and reduce pathological proteins. And there are different intake strategies for different foods. KD is a high fat, low carbohydrate, appropriate protein and nutrients dietary pattern. Neuroprotective mechanisms may involve regulation of GM and metabolite composition, anti-inflammatory, antioxidant, and epigenetic. However, long-term low carbon water intake can cause certain side effects, such as gastrointestinal, weakness, inattention, myodynia and decreased bone density. HbA1c, hemoglobin A1C. CRP, C-reactive protein.

With further research on GM and brain health, GM communicates with the brain through the microbiota-gut-brain axis, intestinal dysbiosis is thought to affect the development of NDs (Kowalski and Mulak, 2019). Therefore, effective reversal or alleviation of intestinal dysbiosis may be a potential strategy to prevent and treat NDs. Regulating GM as a treatment for NDs is a future direction with potential clinical practice. Current approaches based on modulated GM for the treatment of NDs (AD, PD) include diet therapy and fecal microbiota transplantation (FMT; Lorente-Picón and Laguna, 2021; Mohammadi et al., 2019; Westfall et al., 2018; Figure 3).

### Diet therapy

Related studies have proved that diet may affect the occurrence and development of NDs by regulating GM and its metabolite composition, inflammatory response, metabolism, oxidative stress and pathological protein production (Zhang et al., 2020). At present, dietary therapy has shown potential in the treatment of NDs. These include Mediterranean (MedD) and ketogenic (KD) diets (Alfonsetti et al., 2022; Ding et al., 2022; Grochowska and Przeliorz, 2022).

The MedD, a plant-based dietary pattern rich in antioxidants and unsaturated fats. it can change the GM and metabolite composition, modulates systemic inflammation, oxidative stress, metabolic disturbances, neurodegeneration, and cognitive decline (Zhang et al., 2020; Scoditti et al., 2022). It is affected by geographical location and climate (Mattavelli et al., 2022). A cohort study of the health of the Hispanic/Latino community showed that subjects with high adherence to the MedD had better cognitive performance and a lower risk of AD (Moustafa et al., 2022). In addition, high adherence to MedD improved mediotemporal gray matter volume and memory in AD patients, correlated with lower levels of amyloid (Aβ 42/40 ratio), and decreased pTau181 (Ballarini et al., 2021). Also, higher adherence to MedD in midlife was associated with a lower risk of PD (Yin et al., 2021). Therefore, MedD has been recognized worldwide as a good dietary pattern. Olives, nuts, fruits, vegetables and red wine are important components of MedD, because they are rich in nutrients such as unsaturated fatty acids, phenols and vitamins (Petrella et al., 2021; Lefèvre-Arbogast et al., 2022). Treatment of human SH-SY5Y cells transfected with neuronal amyloid precursor protein (APP695) with walnut extract (rich in linoleic acid, oleic acid, α-linolenic acid, and γ-and δ-tocopherol) improved mitochondrial function, increased ATP production, and decreased Aβ1-40 formation. These changes may enhance neurite growth (Esselun et al., 2022).

In addition, dietary polyphenols in MedD have attracted extensive attention due to their neuroprotective properties in NDs (Kaakoush and Morris, 2017; Shishtar et al., 2020; Nargeh et al., 2021). Most dietary polyphenols enter the body and are converted into other microbial metabolites by GM, such as short-chain fatty acids (SFCAs) and phenolic metabolites, thereby delaying the development of NDs (Nargeh et al., 2021; Ticinesi et al., 2022). Dietary polyphenols can exert neuroprotective effects by modulating GM, improving the ubiquitin-proteasome system (UPS) and GM-induced aggregation of pathogenic proteins (Nargeh et al., 2021). Flavonoids belongs to polyphenols, especially anthocyanins and flavono-3-alcohols, as well as flavonoid-rich foods such as berries and red wine, reduce the risk of death in PD patients (X. Zhang et al., 2022). This therapeutic effect may be derived from its inhibition of inflammatory response and reduction of oxidative stress (Zhao Y. et al., 2019). It also can significantly down-regulate the activation of CCAAT/EBPβ/AEP pathway and inhibit the generation of pathogenic proteins in the gut and brain, play an anti-AD effect (Flach et al., 2022). Phosphorylation of α-syn was more pathogenic than α-syn in PD (Penke et al., 2019). Long-term consumption of coffee has a neuroprotective effect on PD, effectively dephosphorylating the pathogenic α-syn by activating subunit protein phosphatase 2A (Yan et al., 2018). Morever, mitochondrial autophagy disorder is also related to NDs (González-Polo et al., 2015). Studies have found that extra virgin olive oil (EVOO) has multiple benefits, such as improving GM composition (Promote the growth of probiotics *Lactobacillus* and *Bifidobacterium*) and cognitive function, antibacterial, anti-inflammatory, antioxidant, lowering blood sugar and insulin resistance (Millman et al., 2021). It can reduce the risk of AD, reduce the abnormal accumulation of Aβ and Tau in AD mouse models, and improve cognition and memory (Millman et al., 2021). Olive oil polyphenols are important components of MedD, which have anti-inflammatory and antioxidant effects (Bucciantini et al., 2021). Studies have shown that olive oil phenolic compounds (olivin) can regulate mitophagy through AMPK/SIRT1/mTOR pathway, and exert antioxidant and anti-cell death effects (Blanco-Benítez et al., 2022). it can also improve the viability of SH-SY5Y cells, reduce the levels of reactive oxide and intracellular free Ca^2+^ induced by proinflammatory protein S100A9 amyloid, and play a neuroprotective role (Leri et al., 2021). Finally, in AD mice fed EVOO, autophagy was induced through AMPK/ UNC-51-like kinase 1 (ULK1) pathway, NLRP3 inflammasome was inhibited to reduce neuroinflammation, clear Aβ, and restore BBB function (Al Rihani et al., 2019). In addition, grapes are also a rich source of polyphenols. Grape extracts have anti-inflammatory, anti-oxidative and protective dopamine neuron functions in PD models *in vivo* and *in vitro* (Ben Youssef et al., 2021). Interestingly, moderate drinking of red wine is also part of MedD, which is rich in polyphenolic compounds (quercetin, myricetin, catechins, tannins, anthocyanidins, resveratrol, ferulic acid). It has a certain antioxidant and neuroprotective effect on AD and PD (Caruana et al., 2016). Finally, MedD may also play a neuroprotective role by modulating GM, and high adherence to MedD is associated with a lower risk of AD and PD. It was found that MedD could increase the level of bacteria associated with AD/PD (2 family, 17 genera, 6 species) and reduce related bacteria (1 family, 17 genera, and 3 species) (Solch et al., 2022). For example, *Lachnoclostridium*, *Parabacteroides* and *Ruminococcus* were increased in AD with MedD, while *Bilophila* and *Streptococcus* were decreased. *Clostridiaceae* 1, *Facealibacterium* and *Prevotella* were increased and *Acidaminococcus* and *Enterococcus* were decreased in PD MedD (Solch et al., 2022). Moreover, MedD can also increase the abundance of beneficial bacteria *Bifidobacterium* and exert anti-inflammatory activity (Lopez-Legarrea et al., 2014). Therefore, MedD show great potential in regulating GM and host homeostasis, and has a good prospect in the field of NDs research.

For KD, it is a high-fat, low-carbohydrate, and recombinant protein diet pattern that has potential neuroprotective effects in NDs, Among them, β-hydroxybutyrate (BHB) and acetoacetate (ACA) are considered to be the most neuroprotective ketone bodies (Li et al., 2020; Gough et al., 2021). The neuroprotective mechanism may involve regulation of GM and metabolism, anti-inflammatory, antioxidant and epigenetic (Gough et al., 2021). A clinical study of 26 AD patients showed significant improvement in quality of life and daily functioning after 12 weeks of modified KD treatment (Phillips et al., 2021). Fortier et al. (2021) also demonstrated that ketone bodies (medium chain triglyceride) can improve mild cognitive impairment in AD patients. In addition, inflammation and Tau aggregation mediated by GM and metabolite changes (such as SCFAs) can be reduced by increasing brain ketone uptake, energy supply, and Aβ clearance, and the same time, it affects the expression of Aβ precursor protein (APP) and α/γ-secretase, thereby improving cognitive impairment, memory, and the ability and quality of daily living (Rebello et al., 2015; Croteau et al., 2018; Nagpal et al., 2020; Neth et al., 2020). A randomized controlled trial of 47 PD patients showed that maintaining KD for 8 weeks was reasonable and safe, with improvements in motor and cognitive function (Phillips et al., 2018). Long-term KD, however, can lead to inadequate carbohydrate intake and certain side effects (known as keto flu), including gastrointestinal distress, weakness, poor concentration, muscle pain, and reduced bone density (especially in children) (Grochowska and Przeliorz, 2022).

In 5XFAD transgenic mice (a mouse model of AD that recapitulates the pathological features of amyloid), 4-month KD can restore the number of neurons and synapses in hippocampus and cortex, reduce microglial activation and amyloid accumulation, reduce neuroinflammation, and improve spatial learning, memory and cognitive function in mice. Shorter KD (2 months) was found to be less effective, while KD in late AD (9 months) had no effect on cognitive improvement (Xu et al., 2022). In addition, based on RNA-seq technology, KD has different effects on neurons and astrocytes, which involve mitochondrial and endoplasmic reticulum function, insulin signal transduction and inflammation related pathways, and is closely related to NDs such as AD (Koppel et al., 2021). Exogenous administration of BHB to AD model mice reduces Aβ plaque formation, increased microglia, apoptosis-associated speck-like protein containing a caspase recruitment domain (Asc) speck and caspase-1 activation and is dependent on inhibition of the NLRP3 inflammasome (Shippy et al., 2020). For PD, a case study showed that after 24 weeks of KD (70% fat, 25% protein, 5% carbohydrate), patients had decreased glycated hemoglobin (HbA1c), CRP, triglycerides, and fasting insulin levels, and improved PD symptoms and anxiety and depression (Tidman, 2022). Morever, KD can also inhibit the reduction of tyrosine hydroxylase (TH) -positive neurons and the activation of microglia in the substantia nigra (SN) of MPTP mice, reduce the levels of proinflammatory cytokines (IL-1β, IL-6, TNF-α), play a neuroprotective and anti-inflammatory role, and alleviate motor dysfunction (Yang and Cheng, 2010). And KD can play a neuroprotective role by regulating glutathione activity against the toxicity of 6-hydroxydopamine (Cheng et al., 2009). Finally, GM including *Lactobacillus*, *Akkermansia*, *Christensenellaceae* and *Enterobacteriaceae* will change after KD intervention, affecting brain function (Varesi et al., 2022). In conclusion, KD may also be a new adjuvant therapy for NDs, which needs further study.

To summarize, Dietary habits interventions have shown a therapeutic effect on pathogenic proteins-induced NDs. The formulation of accurate nutrition plan plays an important role in the prevention and treatment of NDs. In the future, more effective dietary habits can be explored to prevent the occurrence of NDs through large population studies.

### Fecal microbiota transplantation

GM plays an important role in NDs, so remodeling dysregulated GM may be one of the therapeutic strategies. As a new therapeutic approach, FMT transfers fecal microorganisms from healthy donors to the gut of diseased recipients, regulates GM homeostasis, and aims to restore body health (Biazzo and Deidda, 2022). The methods of transplantation include oral live bacteria capsule, upper gastrointestinal intubation, rectal enema and endoscopy (Cold et al., 2021). At present，FMT has been a recognized treatment method for *C*. *difficile* infection (CDI), and is currently the most effective intervention for regulating GM (Surawicz et al., 2013; Varesi et al., 2022). In addition, many clinical experiments have clearly proved that FMT has shown good effects in the treatment of gastrointestinal diseases, metabolic diseases and malignant tumors, and the potential regulatory mechanisms may involve the regulation of GM and metabolite composition, immune response and so on (Qu et al., 2022). Therefore, this section will also focus on reviewing the potential application of FMT in NDs (AD and PD; Table 1).

**Table 1:** Application of FMT in AD and PD.

Disease	Sample	Healthy donor	Transplantation way	Main results	References
AD	A 82-year-old male patient	Patient’s 85-year-old wife	Single infusion of 300 mL FMT (according to Borody method)	CDI was eradicated, the Mini-Mental State Scale (MMSE) score was 29, and memory and mood improved significantly 6 months later	Hazan (2020)
	A 92-year-old femal patient	A 27-year-old healthy male	Due to change of the disease, FMT was performed twice with 60 g fecal suspension, 3 months apart	FMT ameliorated GM dysregulation, with significant differences in alpha diversity (*p* = 0.04), *Bacteroidales*, *Bacteroidia*, *Tannerellaceae*, and *Actinobacteria* were the more enriched taxonomy，involving the pentose phosphate pathway; Orientation, attention and short-term memory improved Short-term side effects: diarrhea, abdominal pain, and fever	Park et al. (2021)
	FMT-young mice (TBI model), FMT-AD mice	3 × Tg-AD (FMT-AD) mice, WT mice	Gavage	FMT-AD can increase the relative abundance of *Muribaculum* and reduce the relative abundance of *Lactobacillus Johnsonii*, significantly impair motor ability, increased microglia/macrophage activation and aggravate neuroinflammation and neurological dysfunction	Soriano et al. (2022)
	ADLP^APT^ mice	Healthy WT mice	After 4 weeks of ABX pretreatment, FMT was administered by gavage for 16 weeks	After FMT, the intestinal barrier was restored, and Aβ plaque burden, Tau pathology, reactive glial activation, and cognitive impairment were alleviated. Reversed aberrant colonic expression of genes associated with intestinal macrophage activity and inflammatory monocytes	Kim et al. (2020)
	APPswe/PS1dE9 transgenic (Tg) mice	Healthy WT mice	Gavage of 0.2 mL/d fecal solution for 4 weeks	After FMT, the GM composition and SFCAs of Tg mice was improved. At the phylum level, *Bacteroidetes* increased, *Proteobacteria* and *Verrucomicrobia* decreased. Cognitive impairment was improved, Tau phosphorylation and Aβ40/42 were decreased, COX-2 and CD11b levels were decreased, and synaptic plasticity (PSD, synaptophysin I) was increased	Sun et al. (2019)
PD	Six patients with PD (age range 47–73, 3 men)	Two healthy male patients (ages 38 and 50)	Colonoscopic infusion of 300 ml fecal suspension was performed in sections (100 ml at the terminal ileum, 100 ml at the cecum and 100 ml along the rest of the colon)	PD-related motor and nonmotor symptoms (including constipation) improved, and one patient developed adverse symptoms and needed to be admitted for observation	Segal et al. (2021)
	Fifteen PD patients (11 males and 4 females, median age 61 years)	Five healthy Chinese Han volunteers (3 males, 2 females, mean age 22 years)	Ten cases were infused by colonoscopy, and 5 cases were infused by nasojejunal tube	FMT relieved both motor and non-motor symptoms of PD (PSQI, HAMD, HAMA, PDQ-39, NMSQ and UPDRS-III scores were significantly decreased (*p* < 0.05)), and colonoscopic infusion seemed to be more effective than nasointestinal FMT. Five cases had adverse reactions: abdominal pain, diarrhea, and flatulence	Xue et al. (2020)
	MPTP PD mouse model	Healthy C57BL/6 mice	Gavage of 1 × 10^8^ CFU/Ml fecal suspension for 7 days	FMT could improve the ecological imbalance of GM, increase the abundance of Firmicutes, decrease the abundance of Proteobacteria, and reduce SCFAs. Striatal DA and 5-HT were increased, TLR4/TNF-α pathway was inhibited, SN microglia and astrocytes activation was decreased, and neuroinflammation was inhibited	Sun et al. (2018)
	Rotenone-induced PD mouse model	Healthy C57BL/6 mice	A total of 100uL fecal suspension, force-feeding method	After FMT, the abundance of *Verrucomicrobia* decreased and *Proteobacteria* increased at the phylum level. At the genus level, *Akkermansia* and *Desulfovibrio* have decreased in abundance and *Barnesiella*, *Butyricicoccus*, *Helicobacter* and *Roseburia* have increased in abundance. Intestinal barrier injury, BBB injury and systemic inflammation were reduced, LPS level *in vivo* was decreased, TLR4/MyD88/ NF-κB pathway was inhibited, and dopaminergic neuron injury was alleviated	Zhao et al. (2021b)
	MPTP PD mouse model	Healthy C57BL/6 mice	1 × 10^8^ CFU/Ml fecal suspension for 7 days，force-feeding method	After FMT, motor dysfunction was improved, fecal SFCAs decreased, α-Syn expression decreased, microglial activation was inhibited, the number of dopamine neurons was increased, and TLR4/PI3K/NF-κB signal transduction was inhibited in SN and striatum	Zhong et al. (2021)

Here we can see the subjects or animal models, healthy fecal donors, fecal microbiota transplantation methods, and the main results after transplantation. CDI, *C*. *difficile* infection; GM, gut microbiota.

A case report of an 82-year-old man with CDI and AD showed a cure of CDI and reversal of AD symptoms after FMT (Hazan, 2020). This was also the case in a recent case report where GM composition, metabolite production, and cognitive function improved after FMT (Park et al., 2021). A study of six men with PD showed improvement in motor and nonmotor symptoms after 6 weeks of FMT in five patients and adverse effects in only one patient (Segal et al., 2021). Another study of 15 PD patients showed similar results, five of whom had gastrointestinal discomfort (abdominal pain, diarrhea, and flatulence; Xue et al., 2020). Therefore, the FMT study of NDs is only in the preliminary stage of animal models. Multiple studies on animal models of AD have shown that after FMT, SCFAs-producing GM increase, Aβ and pTau aggregation and inflammation levels decrease, synaptic plasticity is enhanced, and cognitive function is improved (Sun et al., 2019; Kim et al., 2020; Soriano et al., 2022). A recent study also demonstrated improvements in intestinal barrier, Aβ plaques and neurofibrillary tangles, glial responsiveness, and cognitive impairment in ADLP^APT^ mice (transgenic AD mouse model) after transplantation of fecal microorganisms from healthy mice (Kim et al., 2020). In addition, another study showed that when APP/PS1 transgenic mice were transplanted with GM from AD patients, increased NLRP3 expression in the gut, systemic inflammation, microglial activation, and cognitive impairment were observed. However, GM composition can be improved after transplantation of GM from healthy people or administration of antibiotics (minocycline), and all of the above adverse reactions are improved (Shen et al., 2020).

For PD mice with FMT, GM dysregulation was restored, TLR4/TNF-α inflammatory pathway was inhibited in gut and brain, the activation of SN microglia and astrocytes was decreased, and the levels of striatal dopamine (DA) and 5-HT were increased, which played a role in neuroprotection and alleviating physical injury (Sun et al., 2018). Zhao et al. (2021b) also demonstrated that PD mice treated with FMT significantly restored GM structure, reduced LPS levels in colon, serum and SN, thus inhibited TLR4/MyD88/NF-κB inflammatory signaling pathway, alleviated systemic inflammatory response, restored intestinal barrier and BBB, and played a neuroprotective role. In addition, FMT has been shown to inhibit the expression of α-syn and block the TLR4/PI3K/AKT/NF-κB pathway in the brain (SN and striatum) to exert anti-PD effects (Zhong et al., 2021).

The above contents indicate that FMT has certain feasibility in the future treatment strategy of NDs, but the detailed mechanism still needs to be clarified. Short-term adverse effects (such as gastrointestinal discomfort and fever) have also occurred after FMT, but the exact cause has not been elucidated (Choi and Cho, 2016). All in all, the dysbiosis of GM is closely related to NDs. FMT, as a means to rapidly regulate GM, has great potential for neurotherapy and is economical, which is worthy of further study and provides more options for future prevention and treatment. Morever, in addition to the potential regulatory mechanisms of FMT, future studies need to involve standardized protocols, safety assessments, and increases in sample size and scope.

## Conclusion

Impaired GM structure may be involved in the accumulation of pathogenic proteins in NDS through direct or indirect pathways, and this accumulation may trigger a more severe inflammatory storm through the overactivation of microglia, leading to the development of disease. Overall, GM play a role in the accumulation of pathogenic proteins in the brain, but evidence remains limited. This brain-gut signal crosstalk still requires further study. A large number of clinical trials have shown that dietary therapy and FMT have shown great potential in the treatment of NDs. A stable GM can effectively reduce the occurrence of NDs, but the pathogenesis of each NDs is still different from others. Therefore, the specific mechanism remains to be clarified in the future to tailor a specific diet or GM structure according to different NDs diseases.

## Author contributions

KL and LH initiated and designed the project. SW and WW provided suggestions. CX, XL, and XZ collected the references. All authors contributed to the article and approved the submitted version.

## Funding

This work was supported by the Medical and Health Science and Technology Project of Kunming Health Committee (No. 2021-03-09-001).

## Conflict of interest

The authors declare that the research was conducted in the absence of any commercial or financial relationships that could be construed as a potential conflict of interest.

## Publisher’s note

All claims expressed in this article are solely those of the authors and do not necessarily represent those of their affiliated organizations, or those of the publisher, the editors and the reviewers. Any product that may be evaluated in this article, or claim that may be made by its manufacturer, is not guaranteed or endorsed by the publisher.
